# Polyunsaturated Fatty Acid Balance Modulates Microglial State in a Murine Model of Oxygen-Induced Neovascularization

**DOI:** 10.3390/nu18050749

**Published:** 2026-02-26

**Authors:** Esther S. Kim, Meng-Chin Lin, Cheng-Hsiang Lu, David Casero, Brian Aguirre, Joanne Brown, Olawande Olagoke, Camilia R. Martin, Madhuri Wadehra, Kara L. Calkins, Alison Chu

**Affiliations:** 1 Department of Pediatrics, Olive View-UCLA Medical Center, 14445 Olive View Drive, Sylmar, CA 91342, USA; eskim@dhs.lacounty.gov; 2 Division of Neonatology and Developmental Biology, Department of Pediatrics, David Geffen School of Medicine, University of California-Los Angeles, Los Angeles, CA 90095, USA; mlin@mednet.ucla.edu (M.-C.L.); kcalkins@mednet.ucla.edu (K.L.C.); 3 F. Widjaja Inflammatory Bowel Disease Institute, Cedars Sinai Medical Center, Los Angeles, CA 90048, USAdavid.casero@csmc.edu (D.C.); 4 Department of Pathology Lab Medicine, David Geffen School of Medicine, University of California-Los Angeles, Los Angeles, CA 90095, USAmwadehra@mednet.ucla.edu (M.W.); 5 Department of Gastroenterology, Beth Israel Deaconess Medical Center, Boston, MA 02215, USA; 6 Division of Neonatology, Department of Pediatrics, Weill Cornell Medicine, New York, NY 10065, USA; camilia.martin@med.cornell.edu; 7 Jonsson Comprehensive Cancer Center, University of California-Los Angeles, Los Angeles, CA 90095, USA

**Keywords:** retina, retinopathy of prematurity, oxygen-induced retinopathy, polyunsaturated fatty acids, angiogenesis, hypoxia, microglia

## Abstract

**Background/Objectives:** The retina is enriched in polyunsaturated fatty acids (PUFAs) which are indispensable for normal vision, and recent clinical studies have shown that dietary supplementation of ω-6-and ω-3-polyunsaturated fatty acids (PUFAs) can provide a protective role against retinopathy of prematurity (ROP). Our study aims to understand the mechanisms by which altering ω-6-and ω-3-polyunsaturated fatty acids (PUFAs) in the eye can protect against pathologic retinal neovascularization (NV). **Methods:** We interrogated the effects of endogenous ω-3-PUFA enrichment using transgenic *fat-1* mice which convert ω-6-PUFAs to ω-3-PUFAs in the oxygen-induced retinopathy (OIR) murine model. In the OIR model, mice are exposed to 75% oxygen from postnatal day 7 (P7) to P12, then returned to room air (RA). We used a combination of immunofluorescence, bulk retinal RNA sequencing, and lipid mediator profiling by UHPLC-MS/MS in P17 mouse retinas to identify mechanisms underlying the protective effect against NV seen in *fat-1* mice exposed to OIR. **Results:** *Fat-1* OIR mice were protected against the development of retinopathy, demonstrating 15.1% less vaso-obliteration (75.5% relative reduction) after OIR and a 6.1% reduction in neovascularization (71.8% relative reduction) at P17 (*p* < 0.0001 for both). We found a dampened transcriptional response to OIR in the retina of *fat-1* mice as compared to WT mouse retinas (198 vs. 782 genes, adjusted *p*-value < 0.01). Pathway analyses confirmed these findings, with significant OIR-induced transcriptional shifts in angiogenesis (adjusted *p*-value < 10^−27^), inflammation (adjusted *p*-value < 10^−25^), and microglial activation pathways (adjusted *p*-value < 10^−9^) in WT mouse retina that were not observed in *fat-1* mice. Enrichment scores obtained through the integration of our bulk transcriptomics data with cell-resolved retina data indicate that the protective phenotype observed in *fat-1* mice could be associated with intrinsic differences in microglia cell subtypes between WT and *fat-1* mice. In situ, WT OIR mice demonstrated an increase in Iba1+ microglia compared to WT RA mice, whereas *fat-1* OIR mice showed no difference when compared to *fat-1* RA mice. Three ARA-derived oxylipins, 12-hydroxyeicosatetraenoic acid (12-HETE), prostaglandin D2 (PGD2), and thromboxane B2 (TXB2) demonstrated a pattern of upregulation in WT OIR compared to WT RA, but no upregulation in *fat-1* OIR mice compared to *fat-1* RA. Two EPA-derived specialized pro-resolving mediators and two LA-derived oxylipins were also differentially expressed. **Conclusions:** These findings show that a lower ω-6:ω-3 protects against neovascularization and is associated with attenuation of hyperoxia-induced microglial recruitment and activation, as well as inflammation and angiogenic signaling.

## 1. Introduction

Retinopathy of prematurity (ROP) affects approximately 1 in 3–4 extremely premature infants [1,2]. The incidence of ROP has increased with improved survival of preterm infants [3]. Severe ROP is associated with an increased risk of neurological disability [4,5] and can culminate in retinal detachment and blindness. Laser ablation and anti-vascular endothelial growth factor (VEGF) injections are the only treatments for ROP. However, these treatments are invasive, treat the late phase of ROP, and are associated with increased risk of myopia, cataracts, glaucoma, and retinal thinning [6]. While limiting oxygen exposure mitigates ROP risk, clinicians must balance this risk with adequate oxygen support to sustain tissue oxygenation. Currently, there are no other widely accepted preventative or treatment strategies for ROP [7].

The etiology of ROP is multi-factorial; ROP is spurred on by complex interactions that involve inflammation, oxidative stress, pathological angiogenesis, and altered immune cell recruitment and activation [7,8,9,10,11,12]. The polyunsaturated fatty acids (PUFAs), arachidonic acid (ARA) and docosahexaenoic acid (DHA), representative of ω-6 and ω-3 PUFAs, respectively, play an important role in retinal development and have been implicated in the development and progression of ROP [8,9,10]. ARA and DHA are the most abundant PUFAs in the human retina [11]. PUFAs are structural components of the lipid membrane, alter gene expression by activating nuclear transcription factors [12,13,14,15] and serve as substrates for bioactive lipid molecules, also known as oxylipins and specialized pro-resolving mediators (SPMs) (Supplementary Materials, Figure S1). These molecules regulate diverse processes such as inflammation, oxidative stress, and angiogenesis [13,14].

In preterm infants, circulating PUFA levels decline after birth despite parenteral and enteral nutrition [15,16]. Low ARA and DHA status have been associated with ROP [8,17]. In a multicenter, randomized controlled study of preterm infants at risk of ROP, enteral ARA and DHA supplementation was associated with a 50% decrease in severe ROP (RR 0.50, 95% CI 0.28–0.91, *p* = 0.02) [9]. In a recent meta-analysis sponsored by the American Society for Parenteral and Enteral Nutrition, fish-oil-containing intravenous lipid emulsions, a source of DHA and ARA, were associated with a decrease in the incidence of ROP stage 3 or greater compared to the standard soybean-based lipid emulsion, which is devoid of ARA and DHA (RD; −0.04, 95% CI, −0.08 to −0.01, *p* = 0.02) [18]. Of note, in a recent clinical trial, oral DHA supplementation alone did not protect against ROP [19]. Taken together, these studies suggest that supplementation with both DHA and ARA are important in reducing ROP, but the optimal balance of ω-6:ω-3 is not known and the underlying mechanism of how PUFAs protect against retinal injury in preterm infants is not completely understood.

Mouse models of oxygen-induced retinopathy (OIR) mimic the phases of pathological vascularization in ROP and are used to study retinal angiogenesis. OIR consists of a vaso-obliteration phase fueled by hyperoxia exposure, followed by aberrant neovascularization induced by relative local hypoxia [20,21]. Mouse models of OIR provide experimental proof that PUFAs play a role in ocular disorders [22,23,24]. Connor et al. show that manipulation of retinal ω-6:ω-3 in mice could reduce the pathophysiological effects of hyperoxia exposure on aberrant retinal neovascularization; this group manipulated retinal ω-6:ω-3 using either dietary interventions (10% safflower oil containing 2% ω-3 PUFAS) or using *fat-1* transgenic mice [22]. *Fat-1* mice encode a functional desaturase enzyme that converts ω-6 PUFAs to ω-3 PUFAs [25]. As a result, *fat-1* mice demonstrate a shift towards an ω-3 dominant PUFA profile in the retina, altering the ω-6:ω-3 to 0.45 from 1.22 in wild-type (WT) P17 mice (in the same study, dietary supplementation resulted in a similar retinal ω-6:ω-3 of 0.56). These experiments also demonstrated that both the dietary- and genetically induced reduction in retinal ω-6:ω-3 suppresses tumor necrosis factor-α expression and upregulates expression of DHA-derived SPMs (neuroprotectin D1, resolvin D1, and resolvin E1 (RVE1)) from retinal microglia, the resident macrophages in the neuroretina [22].

Retinal microglia protect against vascular damage by responding to inflammation and injury. In a study by Liu et al., single-cell transcriptome analysis was used to map various microglia populations using a mouse model of OIR [26]. The investigators identified novel highly proliferative and hypermetabolic subsets of microglia in mice exposed to OIR. However, the effects of retinal PUFAs on inflammatory and immunogenic milieu in OIR is still relatively unknown.

Thus, we seek to better characterize how microglia and inflammatory pathways are altered by retinal PUFA balance in murine OIR. In this paper, we utilized the *fat-1* mouse which closely mimics the retinal ω-6:ω-3 observed with dietary ω-3 supplementation in mice and performed RNA sequencing and microscopy studies to better understand how retinal microglia and changes in PUFA balance influence the retinal vascular milieu. We hypothesized that a reduced retinal ω-6:ω-3 ratio would attenuate OIR by altering the balance of microglial states which affect hypoxia-induced inflammation and angiogenesis.

## 2. Materials and Methods

### 2.1. Experimental Approach

Wild-type (WT) mice and *fat-1* transgenic mice were used to investigate the role of altered retinal PUFA balance in oxygen-induced retinopathy (OIR), resulting in four groups: WT RA, WT OIR, *fat-1* RA, and *fat-1* OIR. Mice were exposed to hyperoxic conditions on postnatal day (P) 7 to 12 to induce OIR. On P17, mice exhibited peak neovascularization and experiments were conducted at this time point. Whole-mount imaging was done to first validate the previously reported phenotype of protection against vaso-obliteration and neovascularization in *fat-1* mice compared to WT. Bulk RNA sequencing was done at P17 to identify genes and gene pathways that were differentially regulated in response to OIR in our two strains. Based upon these findings, we did immunofluorescence staining for microglia to assess is situ microglial recruitment and ran liquid chromatography with tandem mass spectrometry to quantitate retinal oxylipins and special resolving mediators to understand how global PUFA-related inflammatory responses are differentially regulated in WT and *fat-1* mice exposed to OIR.

### 2.2. Animal Model

This study was approved by the Animal Research Committee at the University of California, Los Angeles (UCLA), and was in accordance with the National Institutes of Health guidelines. This study was reported in accordance with ARRIVE guidelines. Three transgenic *fat-1* mouse breeding pairs were gifted from the laboratory of Dr. Camilia Martin (Weill Cornell Medicine). This transgenic mouse has the *fat-1* gene of the C. elegans roundworm on a C57BL/6 background. The *fat-1* gene encodes an ω-3 fatty-acid desaturase enzyme that converts ω-6 PUFAs to ω-3 PUFAs [25]. A total of 40 WT mice and 38 *fat-1* mice were used for all experiments.

The UCLA Department of Laboratory Animal Medicine oversaw the colony breeding and maintenance. Mice were housed in standard shoebox-style, corn cob bedding cages enriched with two Nestlets per cage in a 12:12 h light-dark cycle. The room was monitored daily and maintained at 20–26 °C in temperature, 30–70% in relative humidity, and 10–15 room air changes hourly. Genotyping was performed on tail snips to confirm *fat-1* mice, using primers: 5′-CTG CAC CAC GCC TTC ACC AAC C-3′ (forward) and 5′-ACA CAG CAG CAG ATT CCA GAG ATT-3′ (reverse) made through Integrated DNA Technologies (Coralville, IA, USA). Wild-type (WT) C57BL/6 mice (Charles River Laboratories, Wilmington, MA, USA) were used as a control group and had ad libitum access to a standard rodent chow diet (Pico Lab Rodent Diet 20, cat# 5053, Lab Diet, St. Louis, MO, USA). *Fat-1* mice had ad libitum access to Mod TestDiet 58B0 with 10% corn oil (cat#5T9W, Lab Diet, St. Louis, MO, USA) to provide a ω-6 dietary substrate [22]. The diets and their fat content are described in Supplementary Materials, Table S1. *Fat-1* and WT mice were maintained in 12:12 h light–dark cycles.

Sample size was determined based upon previous studies using mouse models of OIR and studies specifically examining retinal outcomes in WT and *fat-1* mice [19,20,21]. The sex of the animals was not considered, as experimental conditions were initiated at P7, prior to sex determination by examination. OIR versus RA experimental groups were randomized, as litters were generated over time. Blinding of the strain and experimental group was done during image scoring or data analysis, as appropriate.

### 2.3. Experimental Model of OIR

Mouse models of OIR were generated as previously published [20,21]. Once pups were delivered, they were set at postnatal day (P) 0.5 on the morning of discovery. *Fat-1* and WT mothers were maintained on their respective diets during gestation and suckling. Mothers and litters were randomly assigned to conditions of OIR or room air (RA). Mothers and litters assigned to the OIR group were placed into a hyperoxia chamber (75% oxygen) on P7 until P12. The hyperoxia chamber (Biospherix Proox model 360, Parish, NY, USA) conditions such as temperature, humidity and mouse health were monitored and checked twice a day while animals were in the chamber. Mice were removed from the hyperoxia chamber on P12 and placed back into RA (21% oxygen). When mothers were in poor health after the stress of the hyperoxia chamber, pups were fostered with a genotype-matched nursing mother. Samples were collected at P17, which represents peak neovascularization (Supplementary Materials, Figure S2).

At the time of dissection, mice were anesthetized with isoflurane. Retinal tissues were dissected and processed for downstream application. Some samples were flash-frozen in liquid nitrogen and stored at −80 °C for processing. Tissues dissected for paraffin embedding were placed in formalin and then stored in 70% ethanol. Samples were collected from 3 to 4 litters at P17 for each condition. Approximately 3–7 mice representing at least 3 litters were analyzed per condition and genotype confirmed, leading to the generation of four groups: WT mice in RA (WT RA), WT mice in OIR conditions (WT OIR), *fat-1* mice in RA (*fat-1* RA), and *fat-1* mice in OIR conditions (*fat-1* OIR) (Supplementary Materials, Figure S2).

### 2.4. Whole-Mount Imaging

After enucleation, retinal samples (WT RA (n = 4, 3 litters), WT OIR (n = 5, 3 litters), *fat-1* RA (n = 3, 3 litters), *fat-1* OIR (n = 5, 3 litters)) were prepared for whole-mount images as previously published [27]. Briefly, eyes were dissected using a surgical microscope (Leica S6D; Leica Microsystems, Durham, NC, USA) and fixed in 4% formaldehyde for 1.5 h at room temperature. Dissected retina cups were washed in phosphate-buffered saline (PBS) and placed in a blocking buffer (20% FBS, 2% goat serum, 0.05% BSA, 1% TritonX-100 in PBS). Retinas were stained with Alexa594-isolectin GS-IB4 (Invitrogen, Carlsbad, CA, USA) and stored overnight at 4 °C in diluent buffer. The next day, retinas were washed with PBS and then mounted onto slides by making incisions at each corner to yield four quadrants. The retinas were mounted with ProLong (Invitrogen, Carlsbad, CA, USA). The flat mounted slides were imaged using an AxioCam CCD digital camera mounted to an inverted epifluorescence microscope (AxioVert 135; Carl Zeiss, Oberkochen, Germany).

### 2.5. Quantification of Vaso-Obliteration and Neo-Vascularization in OIR at P17

Whole-mount images were uploaded and processed in Image J. As previously published, images from P17 were quantified for areas of vaso-obliteration and neovascularization [28]. A blinded scorer quantified areas of vaso-obliteration and neovascularization. The polygonal Lasso tool was used to quantify the total retinal area and area of vaso-obliteration. The quantified number of pixels of vaso-obliteration was then calculated as a percentage of the whole retina. Then the magic wand tool was used to manually outline individual neovascular tufts and unorganized areas of leaky small vessels with a threshold set at 50. The quantified number of pixels of vascularization was then calculated as a percentage of the whole retina.

### 2.6. Immunofluorescence Staining

Eye globes from P17 mice (WT RA (n = 6, 4 litters), WT OIR (n = 6, 3 litters), *fat-1* RA (n = 7, 3 litters), *fat-1* OIR (n = 5, 4 litters)) were enucleated, fixed in formalin for 24 h and then placed into 70% ethanol. Samples were processed for embedding by the UCLA Department of Pathology’s Translational Pathology Core Laboratory (TPCL). Globes were embedded in paraffin and cut into 4 μm sections. One slide was stained with hematoxylin and eosin. Paraffin-embedded retina sections were stained for microglia using anti-Iba1 rabbit monoclonal antibody (Cell signaling, cat #17198 Danvers, MA, USA) at a 1:100 dilution, and for endothelial cells using Lycopersicon esculentum (Tomato) Lectin (Vector Laboratories, cat #FL-1171, Newark, CA, USA) at a 1:850 dilution. Donkey anti-Rabbit 594 (Invitrogen, cat #SA5-10040 Carlsbad, CA, USA) was used for the secondary antibody at a 1:300 dilution. Prolong Gold Antifade Mountant with DAPI (Fisher Scientific, cat # P36935 Hampton, NH, USA) was used as the final step to mount slides and stain nuclei. Slides treated with secondary antibody only were used as controls.

Immunofluorescence (IF) images were captured using the same exposure time and settings with the 40× objective. Acquired images were exported to Image J (version 1.54p; NIH, Bethesda, MD, USA), converted to grayscale, and adjusted using the same histogram-based threshold for each IF staining. The images were then analyzed for Iba1+ or Lectin+ immunoreactive (IR) area percentage.

### 2.7. RNA Sequencing Libraries and Data Analysis

Retina samples (WT RA (n = 3, 3 litters), WT OIR (n = 3, 3 litters), *fat-1* RA (n = 3, 3 litters), *fat-1* OIR (n = 3, 3 litters)) were dissected from WT and *fat-1* mice in conditions of RA and OIR at P17 and flash-frozen. The retinal tissue was suspended in lysis buffer and sonicated. Then the tissue was homogenized using the QiaShredder kit (Qiagen, cat# 79654, Valencia, CA, USA). Once homogenized, RNA was extracted using the Qiagen RNeasy Mini Kit (Qiagen, cat# 74104, Valencia, CA, USA). Samples were then sent to the UCLA Technology Center for Genomics and Bioinformatics for processing. Samples were quantified and tested for RNA degradation and determined adequate for RNA sequencing. Libraries for RNA sequencing were prepared with the KAPA Stranded mRNA-Seq Kit. The workflow consisted of mRNA enrichment and fragmentation, first-strand cDNA synthesis using random priming followed by second-strand synthesis converting cDNA:RNA hybrid to double-stranded cDNA (ds-cDNA), and incorporation of dUTP into the second cDNA strand. cDNA generation was followed by end repair to generate blunt ends, A-tailing, adaptor ligation and PCR amplification. In one lane, different adaptors were used for multiplexing samples. Sequencing was performed on Illumina HiSeq 3000 for SE 1 × 65bp run. Illumina’s SAV v3.0 was used for data quality checking. Illumina Bcl2fastq v2.19.1.403 software was used for demultiplexing. The raw RNA sequencing data unique to this study have been deposited in the National Center for Biotechnology Information (NCBI) Short Read Archive (SRA) with accession code PRJNA1006021. Data from our previous study [27] is available at NCBI’s Gene Expression Omnibus (GSE123945) and was re-analyzed and integrated to incorporate transcriptional changes for P17 WT mice.

Sequence reads were aligned to a genome index that included both genome sequence (GRCm39 mouse primary assembly) and the exon/intron structure of known mouse gene models (Gencode M31 comprehensive genome annotation) using STAR v2.7.10b [29]. Alignment files were used to generate strand-specific gene counts. Independent filtering was applied to remove low-count genes and only protein coding genes were considered for downstream analysis. Expression estimates are reported in units of transcripts per million (TPM) and were computed from the filtered counts matrix after correcting for gene mappable length and per-sample sequencing depth as before [26]. Estimates of cell type proportions for all TPM-normalized samples were obtained using the Gene Expression Deconvolution Interactive Tool (GEDIT [30]) with signatures from the Tabula Muris Reference database [31]. To gain more insight into the microglial enrichment and activation shifts induced by OIR, we performed Gene Set Variation Analysis [32] to estimate enrichment scores on microglia gene signatures from the Mouse Body Atlas and recent high-resolution single-cell studies [26,33,34,35,36].

Unless otherwise noted, count-based and variance-stabilized data (vsd) [37] were used for all ordination, differential and clustering analysis and all figures. Principal component analysis (PCA) was performed using the vsd matrix in R [38]. Differential expression analysis was performed with DESeq2 [37]. For pair-wise tests, genes were classified as regulated when Wald adjusted *p* < 0.05.

Functional analysis of differentially expressed genes was performed with Metascape [39] and enrichment statistics are presented as hypergeometric adjusted *p* values. All plots were generated in R [38] and Matlab (MATLAB, version release 2020b, The MathWorks, Inc, Natick, MA, USA, RRID:SCR_001622).

### 2.8. Ultra-High-Performance Liquid Chromatography with Tandem Mass Spectrometry Measurement of Retinal Oxylipins and Special Resolving Mediators at P17

Retinal samples (WT RA (n = 6, 3 litters), WT OIR (n = 6, 3 litters), *fat-1* RA (n = 6, 4 litters), *fat-1* OIR (n = 5, 3 litters)) were used for targeted analysis of oxylipins and SPMs. Retinal cup samples, ranging from 2 to 8.4 mg (wet weight), were homogenized in 50 µL of filtered 1X PBS. 5 µL of 20 pg/µL internal standard and 945 µL of methanol were added to the 50 µL of homogenized sample, vortexed for 5 s, allowed to sit in ice for 10 min and centrifuged at 15,000× *g* for 30 min at 4 °C. Clear supernatant was transferred to a new tube and dried with a nitrogen evaporator (Organomation, Berlin, MA, USA) at 37 °C. Samples were resuspended in 50 µL of water and methanol (1:1 mixture), vortexed for 5 s, then centrifuged at 15,000× *g* for 1 min at 4 °C. The supernatant was then transferred into amber vials for the oxylipin assay via ultra-high-performance liquid chromatography with tandem mass spectrometry (UHPLC-MS/MS). The extraction and targeted UHPLC-MS/MS analytical procedures were adapted from previously described methods [40]. The UHPLC-MS/MS device comprised UHPLC on the Kinetex® LC column [2.6 µm Polar C18 100 Å 100 × 3.0 mm (Phenomenex, Torrance, CA, USA)], and a tandem mass spectrometry on the hybrid triple quadrupole (QTRAP 6500+) system with multi-component IonDrive™ technology (AB SCIEX LCC, Framingham, MA, USA). Data was acquired in Analyst^®^ Software (version 1.7.2) based on a targeted scheduled multiple reaction monitoring-information dependent acquisition-enhanced production (sMRM-IDA-EPI) method and quantified in SCIEX OS with acquisition parameters shown in Supplementary Materials, Table S2. Lipid mediator concentrations were determined by integrating chromatographic peak areas and calculating analyte-to-internal standard ratios using stable isotope-labeled standards, followed by calibration-based quantification and normalization to retinal tissue wet weight.

### 2.9. Statistical Analysis

All statistical analysis was performed using GraphPad Prism (Version 6, GraphPad Software Inc., La Jolla, CA, USA) except for RNA sequencing data. Kruskal–Wallis testing was used to compare retinal oxylipins and SPMs. In order to assess the potential for false discoveries, we computed the False Discovery Rate (FDR) using the Benjamini/Hochberg method [41]. A *p*-value threshold of 0.05 corresponded to an FDR of 17.5%. Post hoc Dunn’s multiple comparison testing was used to compare two non-parametric data sets. These data are presented as median with interquartile range (25th percentile, 75th percentile) and *p* < 0.05 was used for statistical significance. Outlier exclusion or normalization was not used for any datasets. ANOVA one-way test followed by Bonferroni’s multiple comparisons test was used for flat-mount vaso-obliteration and neovascularization, microglial score and lectin score for immunofluorescence. These data are presented as mean (SEM) and *p* < 0.05 was used for statistical significance. For each condition, 3–7 pups obtained from at least 3 litters were analyzed.

## 3. Results

### 3.1. Fat-1 Mice Demonstrate Attenuation of Pathologic Neovascularization in OIR

**Figure 1:**
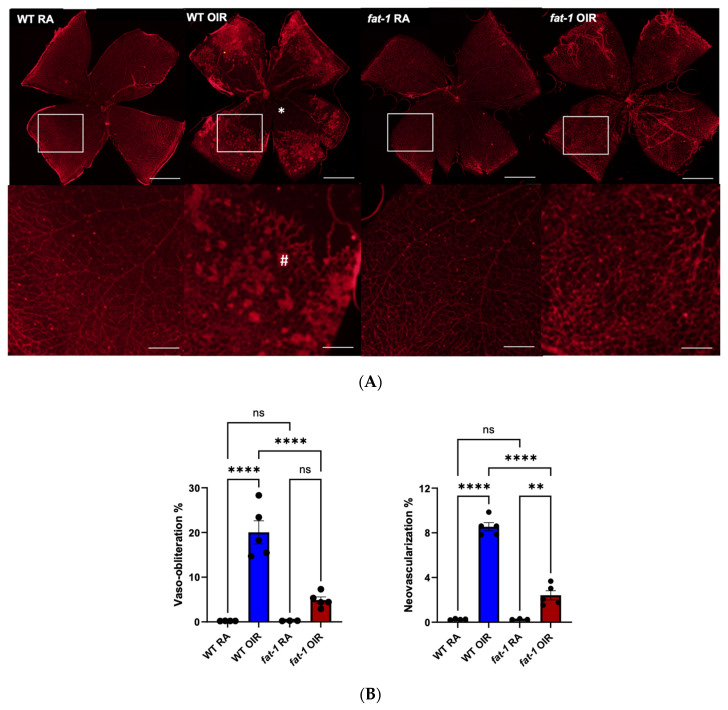
OIR-induced VO and NV at P17 are attenuated in *fat-1* mice compared to WT mice. (**A**) Retinal whole-mount images of WT RA, WT OIR, *fat-1* RA, and *fat-1* OIR mice at P17. WT OIR demonstrated increased central VO (denoted by *) and peripheral NV (denoted by #) compared to WT RA mice, while *fat-1* OIR mice demonstrated significantly less VO or NV compared to WT OIR mice. Retinal whole-mount images shown (top row), with endothelial staining (red) by immunofluorescence. Scale bar = 500 μm. Enlarged inset (white box) images below. Scale bars on inset images = 125 μm. (**B**) Quantification of percentage of VO and NV area represented as mean with SEM. ****: *p* ≤ 0.0001; **: *p* ≤ 0.01, by ANOVA one-way test followed by Bonferroni’s multiple comparisons test. WT RA (n = 4, 3 litters), WT OIR (n = 5, 3 litters), *fat-1* RA (n = 3, 3 litters), *fat-1* OIR (n = 5, 3 litters).

Initially, we validated that *fat-1* mice were protected from pathological neovascularization. As expected, whole-mount imaging of WT RA and *fat-1* RA mice at P17 demonstrated normal retinal vascularization, with no differences between the strains. However, WT OIR mice demonstrated significantly increased central vaso-obliteration at P17 compared to WT RA mice (WT RA 0.2% vs. WT OIR 20.0%, *p* < 0.0001) and concordant increase in peripheral pathological neovascularization (WT RA 0.3% vs. WT OIR 8.5%, *p* < 0.0001). In contrast, *fat-1* OIR mice were protected against the development of retinopathy, demonstrating less vaso-obliteration after OIR (WT OIR 20.0% vs. *fat-1* OIR 4.9%, *p* < 0.0001) and decreased neovascularization (WT OIR 8.5% vs. *fat-1* OIR 2.4%, *p* < 0.0001) compared to WT OIR (Figure 1).

### 3.2. Differential Transcriptional Responses to OIR in WT and Fat-1 Mice

Whole retinal tissue was used for bulk RNA sequencing from WT and *fat-1* mice in RA and OIR conditions. To rule out the effect of differential cell-type composition in gene expression estimates, de-convolution analysis with reference cell types from the mouse body atlas [30] was performed to confirm that all samples from WT and *fat-1* mice were maximally enriched in retina-specific expression, with marginal contribution from other cell type or tissue signatures (Supplementary Materials, Figure S3). In general, we did not detect significant baseline differences in the expression of lineage-defining genes for most retinal cell types [42] between WT and *fat-1* mice, suggesting similar cellular compositions (Supplementary Materials, Figure S4). This was in contrast with the results from principal component analysis of whole-transcriptome data (Supplementary Materials, Figure S4B), which showed a robust segregation of retinal samples from both genotypes in both RA and OIR conditions.

**Figure 2:**
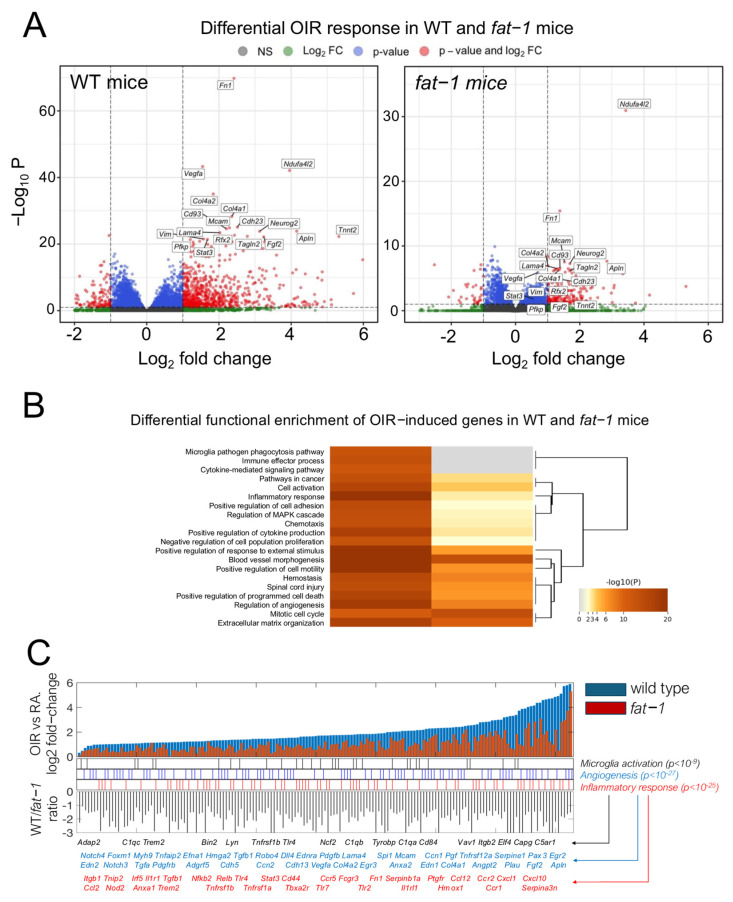
Differential pathway gene expression in WT and *fat-1* mice in response to OIR at P17. (**A**) Volcano plots of global response to OIR in WT (left) and *fat-1* mice (right). Highlighted are genes with maximal response in WT mice, with dampened or not significant responses in *fat-1* mice. The *x*-axis represents log2 fold changes between OIR and RA mice and has the same scale in both plots. The *y*-axis represents −log10 adjusted *p*-values. Note the scale is different for both genotypes given the more marked statistical significance in WT mice. (**B**) Functional enrichment results of whole-retina RNA sequencing. Significantly regulated genes in response to OIR as compared to RA were strongly enriched in inflammation, angiogenesis, and microglial activation pathways in wild-type (WT) mice (left column in heatmap). These same pathways are less significant in *fat-1* mice (right column). (**C**) Differential regulation of key pathway genes in response to OIR in WT and *fat-1* mice. Shown are results for genes that exhibited strong upregulation in WT mice and dampened or no upregulation in *fat-1* mice in response to OIR. Top: bar plot of log2 fold changes (y axis) in OIR conditions as compared to RA for P17 mice in both genotypes. Genes are sorted by increasing fold change in WT mice (x axis). Middle: position of genes annotated in pathways significantly associated with OIR upregulation. Selected gene names are shown below. Bottom: bar plot of WT vs. *fat-1* log2 fold-change ratios. WT RA (n = 3, 3 litters), WT OIR (n = 3, 3 litters), *fat-1* RA (n = 3, 3 litters), *fat-1* OIR (n = 3, 3 litters).

However, we observed major differences in the global response to OIR between genotypes. Differential expression revealed a marked response to OIR in WT, with a dampened or less significant effect of OIR in *fat-1* mice (Figure 2A). Functional enrichment analysis demonstrated a distinct impact of OIR in inflammatory, angiogenic, and microglial activation pathways (Figure 2B). While we noted a distinct functional enrichment for genes with strong OIR-induced upregulation in WT mice, we also observed a moderate or absent response to OIR in *fat-1* mice in pathways involving inflammatory response (adjusted *p*-value < 10^−25^), angiogenesis (adjusted *p*-value < 10^−27^), and microglial activation (adjusted *p*-value < 10^−9^) (Figure 2C). 

### 3.3. Fat-1 Mice Have Attenuated OIR-Induced Microglial Recruitment and Activation and Differential Microglia Subset Enhancement Compared to WT Mice

**Figure 3:**
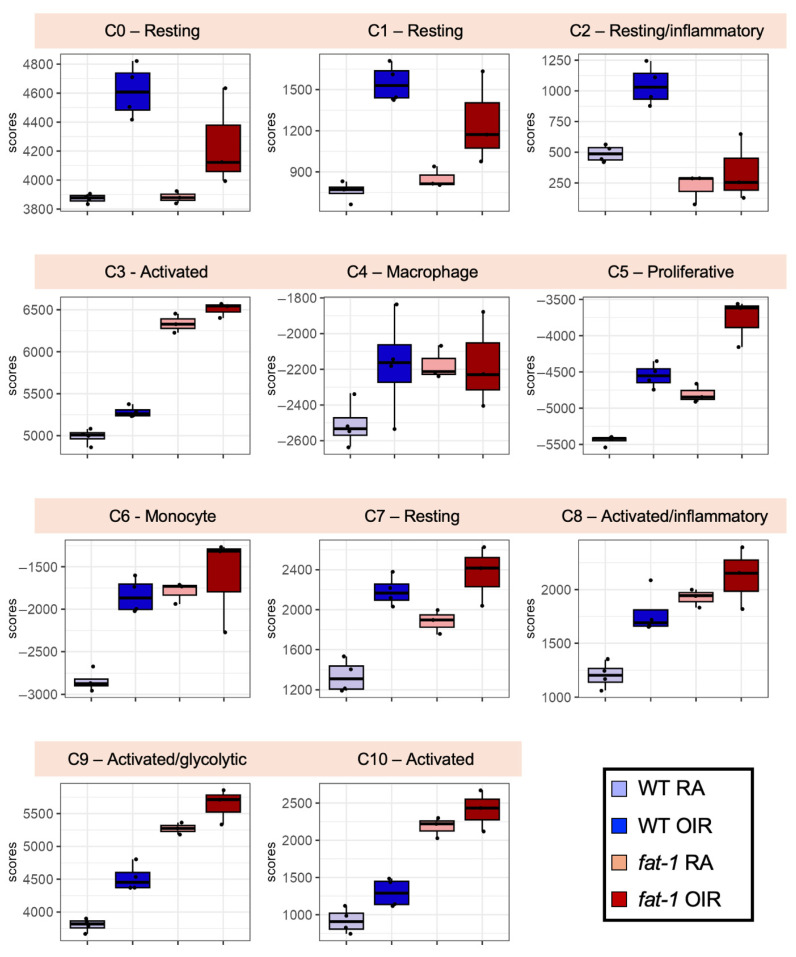
Microglial enrichment scores in WT and *fat-1* whole-retinal samples in RA and OIR conditions. Enrichment scores of WT and *fat-1* whole-retinal samples by bulk RNA-Seq on a panel of microglia subtype signatures derived from single-cell profiling by Liu et al. Cluster numbers (C0 through C10) corresponding to microglia subtypes and their phenotypic annotations are as defined in the publication.

Microglial activation and immune processes were among the top differentially altered pathways in WT mice but less impacted in *fat-1* mice (Figure 2). We therefore performed enrichment analyses using fine-grained microglia signatures collected from high-resolution single-cell studies [26,33,34,35,36] to understand if WT and *fat-1* mice are associated with distinct microglial activation phenotypes (Figure 3). In particular, using marker genes of resting and activated microglia subtypes in WT P17 RA and OIR mice defined by Liu et al. [26], we observed that OIR induced an enrichment in proliferative phenotypes in both WT and *fat-1* mice (microglia subtype 5, Figure 3). Generic monocyte and resting microglia subsets (microglia subtypes 0, 1, 2, 4, 6, and 7) had significantly higher induction by OIR in WT mice compared to *fat-1* mice. Higher enrichment in response to OIR for subtypes 2 and 8, representing pro-inflammatory resting and activated microglia, was predominantly observed in WT mice. Interestingly, *fat-1* mice demonstrated baseline enrichment compared to WT mice in precursor and activated microglial subtypes (subtypes 3, 5, 9 and 10) in both RA and OIR conditions. Of note is that, compared to WT mice, *fat-1* mice show an attenuated response in genes associated with glycolytic microglia (Supplementary Materials, Figure S5A), with an attenuated response to OIR in *Slc16a3*, *Mt1*, *Gpi1*, and *Aldoa* genes. In addition, WT mice showed clear upregulation of injury-related microglia genes [33] in response to OIR (notably *Mrc1*, *Ccr1*, *Ms4a6c*, *Ms4a6b*, *Ms4a6d*, *Tmem176a*), indicative of a robust microglial injury response not observed in *fat-1* mice (Supplementary Materials, Figure S5B).

**Figure 4:**
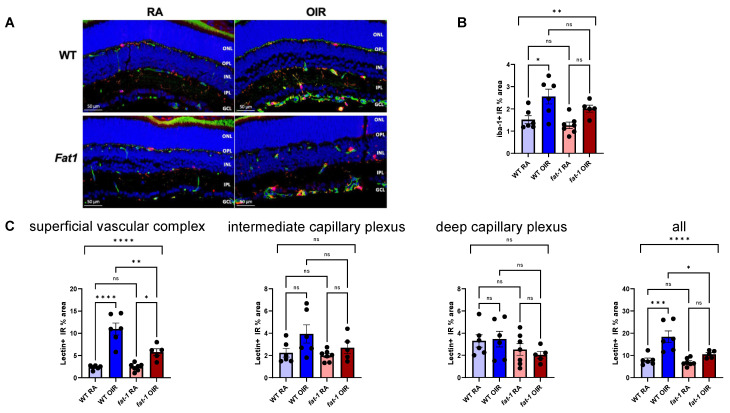
Microglia in WT and *fat-1* murine retina at P17 in RA and OIR conditions. (**A**) Detection of microglia in murine retina samples at P17 with anti-iba1 antibody (red) by immunofluorescence. Blood vessels indicated by anti-lectin antibody (green). ONL, outer nuclear layer; OPL, outer plexiform layer; INL, inner nuclear layer; IPL, inner plexiform layer; GCL, ganglion cell layer. Scale bars = 50 μm. Quantification of staining for iba-1 (**B**) and lectin by plexus and in total (**C**). Bars represent mean (SEM) in the amount of immunoreactive (IR) percent area. ****: *p* ≤ 0.0001; ***: *p* ≤ 0.001; **: *p* ≤ 0.01, *: *p* < 0.05, by ANOVA one-way test followed by Bonferroni’s multiple comparisons test. WT RA (n = 6, 4 litters), WT OIR (n = 6, 3 litters), *fat-1* RA (n = 7, 3 litters), *fat-1* OIR (n = 5, 4 litters).

We sought to characterize microglial recruitment in situ using immunofluorescence staining. The baseline Iba1 microglial scoring at P17 in *fat-1* RA mice was similar to WT RA mice (WT RA 1.5 ± 0.2 vs. *fat-1* RA 1.3 ± 0.1, n = 6–7/group; *p* > 0.05). WT OIR mice demonstrated an increase in microglial Iba1 score compared to WT RA mice, particularly near the superficial vascular plexus, (WT RA 1.5 ± 0.2 vs, WT OIR 2.6 ± 0.3; n = 6/group; *p* < 0.05), whereas the *fat-1* OIR mice showed no significant differences in their microglial Iba1 score compared to *fat-1* RA mice (*fat-1* RA 1.3 ± 0.1 vs. *fat-1* OIR 2.0 ± 0.2; n = 5–6/group; *p* > 0.05) (Figure 4A,B). As expected, the vascular staining was increased in the WT OIR group compared to the WT RA group in the superficial vascular plexus (WT RA 2.3 ± 0.2 vs. WT OIR 11.0 ± 1.3; n = 6/group; *p* ≤ 0.0001), but to a much lesser extent in the *fat-1* OIR group compared to *fat-1* RA (*fat-1* RA 2.4 ± 0.3 vs. *fat-1* OIR 5.8. ± 0.8; n = 5–7/group; *p* < 0.05). We did not observe differences in vascular staining among the four groups in the intermediate or deep vascular plexuses (*p* > 0.05 for all). Lastly, we observed that the microglia largely co-localized along the retinal vascular plexuses.

### 3.4. Fat-1 Mice Exhibit Dampened Upregulation of ARA-Derived Oxylipins and DHA-Derived Resolvins in Response to OIR, Compared to WT Mice

**Figure 5:**
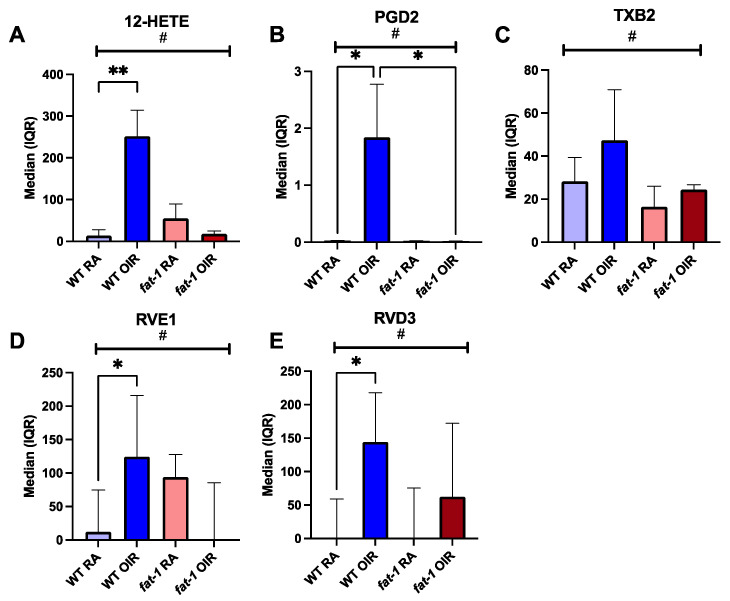
Retinal oxylipins and SPMs at P17 in WT and *fat-1* mouse retinas in RA and OIR conditions. Concentrations of lipid mediators quantified by targeted UHPLC-MS/MS-based profiling. Concentrations (pg/mg) of ω-6 derived (**A**) 12-Hydroxyeicosatetraenoic acid (HETE) (**B**) ProstaglandinD2 (PGD2) and (**C**) Thromboxane B2 (TXB2) in WT RA, WT OIR, *fat-1* RA, and *fat-1* OIR mice. *Fat-1* mice had decreased or stable concentrations of 12-HETE, PGD2, and TXB2 when exposed to OIR, compared to RA, whereas WT mice showed elevated concentrations in response to OIR compared to RA. Elevated concentrations of ω-3-derived SPMs (**D**) RVE1 and (**E**) RVD3 in WT OIR when compared to WT RA. *Fat-1* OIR RVE1 and RVD3 concentrations were unchanged compared to *fat-1* RA. ^#^ *p* < 0.05 when comparing all four groups by Kruskal–Wallis for all shown. * *p* < 0.05 and ** *p* ≤ 0.01 analysis by Dunn’s multiple comparisons test. WT RA (n = 6, 3 litters), WT OIR (n = 6, 3 litters), *fat-1* RA (n = 6, 4 litters), *fat-1* OIR (n = 5, 3 litters).

We quantified downstream bioactive lipid molecules at P17 in the setting of OIR using targeted UHPLC-MS/MS oxylipin and specialized pro-resolving mediator profiling. Seven of 28 metabolites showed significant differences among the groups. Three ARA-derived oxylipins, 12-hydroxyeicosatetraenoic acid (12-HETE), prostaglandin D2 (PGD2), and thromboxane B2 (TXB2) demonstrated a pattern of upregulation in WT OIR compared to WT RA, but no upregulation in *fat-1* OIR mice compared to *fat-1* RA. These oxylipins were significantly different when all four groups were compared (*p* < 0.02, by Kruskal–Wallis for all). In WT mice, 12-HETE was greatly increased in conditions of OIR when compared to RA (WT RA 0.02 pg/mg, IQR: 0.01–20.92 vs. WT OIR 252 pg/mg, IQR: 82–314; *p* = 0.002). In contrast, in *fat-1* OIR mice, there was no difference in 12-HETE compared to *fat-1* RA (*fat-1* RA 31.04 pg/mg, IQR: 0.03–86.87 vs. *fat-1* OIR 16 pg/mg, IQR: 5–32); *p* > 0.99) (Figure 5A). PGD2 demonstrated a similar pattern. PGD2 was increased in WT OIR compared to WT RA and compared to *fat-1* OIR (WT RA 0.018 pg/mg, IQR: 0.014–0.032 vs. WT OIR 1.12 pg/mg, IQR: 0.02–3.77; *p* = 0.04) (WT OIR 1.12 pg/mg, IQR: 0.02–3.77 vs. *fat-1* OIR 0.019 pg/mg, IQR: 0.018–0.020; *p* = 0.02) (Figure 5B). TXB2, an inactive metabolite of thromboxane A2 (TXA2), was differentially expressed when four groups were compared, with the highest expression in WT OIR (WT RA 28 pg/mg, IQR: 15–39; WT OIR 47 pg/mg, IQR: 27–71; *fat-1* RA 16 pg/mg, IQR: 11–26; *fat-1* OIR 24 pg/mg, IQR: 12, 27; *p* = 0.015) (Figure 5C). Two LA-derived oxylipins, 9-hydroxyoctadecadienoic acid (9-HODE) and 13-Hydroxyoctadecadienoic acid (13-HODE) demonstrated downregulation in WT OIR compared to WT RA, but upregulation in *fat-1* OIR compared to *fat-1* RA (*p* = 0.03 for both).

Interestingly, two ω-3-derived SPMs exhibited a differing pattern. RVE1, a SPM derived from EPA, was increased in WT OIR when compared to WT RA (WT RA 12.09 pg/mg, IQR: 0.02–75.52; WT OIR 125 pg/mg, IQR: 88–216; *p* = 0.03) (Figure 5D). Likewise, resolvin D3 (RVD3), a DHA-derived SPM, was increased in conditions of OIR when compared to RA in WT mice (WT RA 0.02 pg/mg, IQR: 0.01–59.08 vs. WT OIR 145 pg/mg, IQR: 80–218; *p* = 0.02) (Figure 5E). In contrast, RVE1 and RVD3 concentrations in *fat-1* mice in OIR versus RA were not different, indicating an attenuated or no response in these resolvins in *fat-1* mice under OIR conditions. There was a significant difference in both RVE1 and RVD3 when comparing all groups (*p* = 0.02 by Kruskal–Wallis), though notably wide variability in all groups (Figure 5D,E).

Other oxylipin and SPM measurements are shown in Supplementary Materials, Table S3.

## 4. Discussion

The pathophysiology of ROP is multifactorial. Extremely premature infants who develop chronic lung disease secondary to prolonged intubation and oxygen exposure are at high risk of severe ROP [1]. Local hyperoxia induces inflammatory and oxidative cascades that dysregulate angiogenesis and immune responses, contributing to ROP [43,44]. While supplementation of ARA and DHA in premature infants at risk for ROP appears to mitigate ROP outcomes [9], the optimal PUFA supplementation strategy and how ω-6:ω-3 protects against ROP is unknown.

Previous work reported a decrease in retinal ARA and an increase in retinal DHA in *fat-1* mice at P17 compared to WT mice in normoxic conditions, analogous to the retinal PUFA composition that is seen after enteral PUFA supplementation in mice [22]. Here, we validated that *fat-1* OIR mice are protected against vaso-obliteration and neovascularization at P17, the sine qua non of OIR [22]. Our study builds upon prior studies which focus on specific genes/pathways, by instead using broad transcriptomic and lipidomic analyses to conduct an unbiased evaluation of tissue-level responses after alteration of PUFA balance in the retina. Our results suggest that the protective effect of lower retinal ω-6:ω-3 may be partially mediated by altered expression of ARA-derived oxylipins and ω-3-PUFA-derived SPMs in association with reduced activation of pro-inflammatory microglial subsets.

In our study, bulk RNA sequencing of mouse retina revealed that OIR dramatically altered the retinal transcription of genes involved in angiogenesis, inflammation, and immune response pathways in WT mice, but this transcriptional shift in response to OIR was attenuated in *fat-1* mice. In response to OIR, *fat-1* mice showed weaker activation of several angiogenesis pathways relative to WT mice. In our study, the upregulation of *VEGFa*, the main isoform implicated in the pathologic angiogenesis of ROP [45], was dampened in *fat-1* mice compared to WT mice. We also found multiple other angiogenic signaling genes were significantly more upregulated in WT OIR than in *fat-1* OIR (including *Pdgfb*, *Col4a2*, *Edn1*, *Ccn1*, *Angpt2*, *Socs3*, *Fgf2*, *Edn2*, *Aplnr*, *Sox9*, *Itgb1*, *Tgfa*, *Pdgfrb*, *Mmrn2*, *Elk3*, *Adgrf5*, *Robo4*, *Itga5*, *Ednra*) compared to their RA counterparts. These genes have been implicated in retinal vascular development and other pathologic angiogenic eye disorders such as diabetic retinopathy, age-related macular degeneration, choroidal neovascularization, and ischemic retinopathy [46,47,48,49,50,51,52,53,54,55,56,57,58,59], underscoring the strong association between ω-6:ω-3 and regulatory angiogenic networks.

An inflammatory milieu, spurred on by noxious factors such as infection or hyperoxia, has been shown to induce ROP [42,60,61]. Our transcriptome analysis identified significant upregulation of many pro-inflammatory genes in WT OIR mice, but with a dampened response in *fat-1* OIR mice. Within the inflammatory pathway, proinflammatory genes such as *Nfkb2*, *Tlr2*, *Tlr7*, *Cxcl1*, *Cxcl10*, *Ccl2*, and *Ccl12* were substantially increased in OIR vs. RA WT mice compared to *fat-1* mice exposed to the same conditions. *Cxcl1*, *Cxcl10*, *Ccl2* and *CCl12* have been implicated in vascular permeability and immune recruitment in the context of retinal injury [62,63,64,65]. The overexpression of *Tlr2*, encoding a toll-like receptor that plays a role in pathogen recognition, could also be part of an innate response to OIR-induced retinal injury [66]. In agreement with our results, TLR2 has been reported to be inhibited by ω-3 PUFAs in macrophage cell culture [66,67,68] and in animal models of rosacea [69].

In our study, nuclear factor kappa beta (NF-κβ) and tumor necrosis factor (TNF) receptors were also significantly upregulated in WT OIR mice with little upregulation in *fat-1* OIR mice. Other studies corroborate this finding: in mice fed an ω-3-rich diet, there was suppression of TNF-α, which is secreted from retinal microglia [22]. Microglia are resident macrophages of the retina and regulate retinal vascular development [70]. Although traditionally considered immune-privileged, microglia, a subset of myeloid cells, play a crucial role in regulating and maintaining the microenvironment in response to injury, hypoxia, neuroinflammation, degeneration, or aging. It has been shown that in response to these stimuli, microglia can undergo distinct activation processes including rapid proliferation, migration to affected areas, phagocytosis of cellular debris, and the release of cytokines, chemokines, or proangiogenic factors that influence neighboring retinal cells [26,33,71,72,73,74]. Liu et al. [26] proposed that retinal microglia play a protective role in the retina; global retinal microglia inhibition via clondronate administration before hyperoxia exposure increases vaso-obliteration and neovascularization. This highlights that differing microglial states, which can be altered by noxious stimuli in the local retinal environment, fuel important injury responses that can serve to worsen or mitigate retinopathy. By leveraging single-cell signatures reported by Liu et al. [26] and integrating our transcriptome-sequencing data from the whole retina in WT and *fat-1* mice, we can infer a retina-wide shift to activated and proliferative microglia in response to OIR.

Based on bulk RNA sequencing signatures, both WT and *fat-1* mice demonstrate a shift to proliferative microglia in OIR conditions. However, WT mice demonstrate a more marked shift in certain “resting” microglial clusters to OIR conditions compared to *fat-1* mice. Interestingly, *fat-1* mice show a higher baseline enrichment in “activated/inflammatory” microglial signatures in RA, which may explain why these signatures experienced an enhanced or “catch up” response to OIR in WT mice. These subtypes were described by Liu et al. [26] as intermediate and precursor microglia states that can differentiate into different functional subtypes in response to external insults, thus driving a reversible metabolic activation after OIR. Enrichment of these distinct subsets in *fat-1* mice suggests that a lower ω-6:ω-3 confers a higher phenotypic plasticity in response to OIR. When we took a closer look at these microglial clusters, microglial injury and the glycolytic microglial response were dampened in *fat-1* mice in response to hyperoxia compared to WT. Microglia shift from oxidative phosphorylation towards glycolysis in inflammatory conditions such as diabetic retinopathy [35], producing acetyl-CoA which can modify gene expression towards pro-inflammatory responses. This glycolytic shift is encompassed within a larger microglial metabolic reprogramming which has been implicated in neurodegenerative disorders such as Alzheimer’s disease, some of which also affect the retina [75]. This pattern suggests that a decreased ω-6:ω-3 attenuates OIR-induced microglial pro-inflammatory activation in certain microglial subsets. Interestingly, in WT mice, this shift in response to OIR is accompanied by recruitment of microglia in situ, whereas *fat-1* mice do not demonstrate microglial recruitment. In our data, altered retinal PUFA balance is associated with differential microglial activation via shifts in glycolysis, which would be a novel potential mechanism not previously identified in OIR.

Given the interesting findings regarding differential microglial “shifts” in response to OIR between WT and *fat-1* mice, we seek to identify a potential mechanistic link between PUFA balance, microglial states, and the inflammatory microenvironment within the neuroretina in response to OIR. Amongst the many potential effects that PUFAs may have in the eye, the bioactive PUFA-derived mediators known as SPMs and oxylipins are known to affect microglial activation [76]. Resolvins (resolution phase interaction products) are bioactive mediators derived from EPA and DHA [22] that promote inflammatory resolution [77,78]. They have been described to have a protective effect against diabetic retinopathy in animal models [79,80] and aberrant hyperglycemia-induced angiogenic signaling in vitro [81]. RveE1 inhibits the activity of NF-κβ and the subsequent production of proinflammatory cytokines like TNF and Il1b [82], which are produced by microglial subtypes such as clusters 1 and 2 described by Liu et al. [26] (Figure 3). In our study, in WT mice, hyperoxia generated a robust RVE1 and RVD3 response to possibly help curb retinal inflammation. We also observed an attenuated increase in RVD3 and a decrease in RVE1 in *fat-1* OIR mice compared to WT OIR mice. Connor et al. [22] demonstrate a similar pattern though in different SPMs; retinal resolvin E2 (RVE2), a biosynthetic marker for RVE1, decreased at P17 in WT mice fed an ω-3 rich diet after hyperoxia exposure compared to normoxia conditions. Our SPM evaluation is broader than that previously described in *fat-1* OIR mouse studies, in that we also evaluate ω-6-derived oxylipins. We believe this is important as recent clinical studies show clearly that both ω-6 and ω-3 PUFAs play a role in modulating ROP.

In addition to the changes observed in ω-3-derived SPMs, we observed changes in ARA-derived oxylipins. Oxylipins are generated via oxygenation of PUFAs, suggesting that they may act as a sensor of aberrant oxygen exposure. 12-HETE and PGD2, two ARA-derived oxylipins, were increased in the WT OIR group vs. WT RA group. However, this response in 12-HETE and PGD2 was not observed in *fat-1* mice in response to OIR. 12-HETE is derived from ARA via 12-lipoxygenases (12-LOX) [83]. There is evidence that 12-LOX and its product, 12-HETE, play a role in regulating inflammatory and angiogenic processes that underlie diabetic retinopathy [84]. 12-HETE has also been shown to increase VEGF expression in human and porcine vascular smooth-cell cultures [85]. In a human metabolomics study of diabetic retinopathy, serum 12-HETE was a better predictive marker for diabetic retinopathy than HgbA1c, a standard test for assessing glycemic control [86]. PGD2 is generated via the enzyme cyclooxygenase; it is abundant in the choroid [87]. In our study, OIR increased PGD2. PGD2 has been shown to mediate choroid involution and endothelial cell apoptotic death induced by OIR [88,89]. We speculate that hyperoxic injury induces ARA-derived oxylipins such as PGD2 that promote aberrant angiogenesis and inflammation, but a pro-resolution response is mounted by the metabolism of ω-3 PUFAs (RVD3, RVE1) in WT mice. Of note, two of our metabolites that differed in our experimental groups, 9-HODE and 13-HODE, did not follow this trend: they both decreased in WT OIR conditions but increased in *fat-1* OIR compared to RA. These oxylipins are known to be biomarkers of oxidative stress and to be involved in inflammatory processes, but their roles in the retina have not been characterized. In our study, a decreased retinal ω-6:ω-3 in the setting of OIR attenuates both the hyperoxia-induced increase in ARA-derived oxylipins and the EPA- and DHA-derived resolvin-mediated pro-resolution response.

Our study has limitations. Our study was not powered to detect potential sex differences, which are known to have an effect in lipid metabolism [90]. Although the proposed mechanism in our study is derived from in vivo bulk RNA sequencing and we report a complete segregation of WT and *fat-1* retinas at the transcriptional level, we limited our discussion on OIR-induced changes to the more significant observations due to limited sample sizes and within-group variability. Also, shifts in microglial subtypes are inferred rather than directly measured, and future studies should include experiments allowing for single-cell resolution, such as single-cell RNA sequencing, functional microglial assays (e.g., metabolic flux, cytokine production), or validating the effect of manipulating specific pathways in co-culture assays. Lastly, because *fat-1* mice skew ω-6:ω-3, we cannot disentangle the effect of individual PUFAs on the retinal phenotype. Though prior published studies demonstrate that the retinal tissue concentrations seen in *fat-1* mice are comparable to dietary interventions in mice [22], our observed effects cannot be automatically extrapolated to nutritional interventions. Moreover, using this mouse model limits us in the PUFA ratio that can be achieved in the tissue, whereas animal studies using dietary manipulation may allow skewing of ω-6:ω-3 to identify an “optimal” balance [91]. However, our findings remain important, as they can guide future studies with varying dietary supplementation in mouse models beyond just evaluating the phenotypic output of vaso-obliteration and neovascularization. Nonetheless, the challenge remains how these manipulations in mice can be translated to clinical care, given that, in mice, retinal PUFA levels can be measured after dietary measurements, but in humans, how dietary manipulations change retinal PUFA levels cannot be measured. We utilize these animal models to better inform our understanding of what may be happening in the eye, in a manner that would be challenging or impossible in clinical studies. Our hope is that these studies provide mechanistic insights that better inform preventative and therapeutic strategies of PUFA supplementation in the clinical care of preterm infants at risk for ROP.

## 5. Conclusions

Our study demonstrates that a decreased ω-6:ω-3 protected against aberrant retinal vascularization induced by OIR. This protection may be mediated by complex interactions of PUFAs and generation of downstream bioactive lipid molecules that dampen inflammation, aberrant angiogenesis, and pathological microglial recruitment and activation after hyperoxia exposure. Further research is needed to precisely understand how PUFAs regulate retinal development, function, and long-term vision in the preterm infant.

## Data Availability

The raw RNA sequencing dataset generated and/or analyzed during the current study has been deposited in the National Center for Biotechnology Information (NCBI) Short Read Archive (SRA) with access code PRJNA 1006021. Data from our previous study [26] is available at NCBI’s Gene Expression Omnibus (GSE123945).

## Supplementary Materials

The following supporting information can be downloaded at: https://www.mdpi.com/article/10.3390/nu18050749/s1, Figure S1: Metabolism of polyunsaturated fatty acids and their downstream products; Figure S2: Murine model of oxygen-induced retinopathy (OIR) and experimental design; Figure S3: Deconvolution analysis of whole-retina RNA-Seq samples; Figure S4: Gene expression heatmap of cell-type markers for retinal cell population; Figure S5: Bulk RNA-Seq gene expression of glycolysis and injury-related microglia genes; Table S1: Mouse Diets; Table S2: UHPLC-MS/MS parameters for targeted oxylipin and specialized pro-resolving mediator analysis; Table S3: Retinal oxylipins quantified by targeted UHPLC-MS/MS and their parent polyunsaturated fatty acids (PUFAs) at P17.

## Abbreviations

The following abbreviations are used in this manuscript:

12-HETE	12-hydroxyeicosatraenoic acid
ARA	arachidonic acid
DHA	docosahexaenoic acid
EPA	eicosapentaenoic acid
IF	immunofluorescence
NV	neovascularization
NF-κβ	nuclear factor kappa beta
OIR	oxygen-induced retinopathy
P	postnatal day
PGD2	prostaglandin D2
PUFA	polyunsaturated fatty acid
RA	room air
ROP	retinopathy of prematurity
RVE1	resolvin E1
RVE2	resolvin E2
RVD3	resolvin D3
SPM	specialized pro-resolving mediators
TNF	tumor necrosis factor
TXB2	thromboxane B2
UHPLC-MS	Ultra-High-Performance Liquid chromatography-mass spectrometry
VEGF	vascular endothelial growth factor
VO	vaso-obliteration
WT	wild-type
